# Supplementary material for: Research on the impact of User-Generated Content (UGC) in shaping the purchase behavior of environmentally friendly products and the moderatings role of brand reputation
Source: PLoS One. 2025 Nov 21;20(11):e0337193. doi: 10.1371/journal.pone.0337193 (PMC12637987; doi:10.1371/journal.pone.0337193)
Supplement: Attachment — Submitted filename: Response to Reviewers.docx [file pone.0337193.s002.docx]

**Manuscript title:** Research on the Impact of User-Generated Content (UGC) in Shaping the Purchase Behavior of Environmentally Friendly Products and the Moderating role of Brand Reputation

**Manuscript Number:** PONE-D-25-13451

| **No.** | **Journal Requirements** | **Authors’ explanations** |
| --- | --- | --- |
|  | Please ensure that your manuscript meets PLOS ONE's style requirements, including those for file naming | The authors have adjusted the manuscript according to the journal’s style and formatting requirements and renamed the revised version as instructed. |
|  | You indicated that you had ethical approval for your study. In your Methods section, please ensure you have also stated whether you obtained consent from parents or guardians of the minors included in the study or whether the research ethics committee or IRB specifically waived the need for their consent. | The authors have stated the required statements in method sections:  Participation in the study was entirely voluntary, and all participants provided verbally informed consent before taking part. Prior to responding, participants were fully informed about the study’s objectives, the scope of their participation, and their right to withdraw at any time without consequences. Verbal consent was documented by the survey administrators before data collection commenced. Among the participants, two respondents were under the age of 18. For these cases, parental or guardian consent was obtained prior to data collection. The study was approved by the Ethics Committee of Saigon University, which confirmed that the research procedures complied with institutional ethical standards and national regulations for human participant research. |
|  | We note that the grant information you provided in the ‘Funding Information’ and ‘Financial Disclosure’ sections do not match.  When you resubmit, please ensure that you provide the correct grant numbers for the awards you received for your study in the ‘Funding Information’ section. | The authors have corrected Funding information as follows:  This research is funded by Saigon University, Ho Chi Minh City, Vietnam, under the Scientific Research Contract No. 369/HĐ-ĐHSG, dated May 5, 2025.  Project code: CS.2025.A2.003  Project title: “The Impact of User-Generated Content (UGC) on the Purchase Behavior of Environmentally Friendly Products and the Moderating Role of Brand Reputation – Evidence from the Hospitality Industry in Vietnam” |
|  | We note that you have indicated that there are restrictions to data sharing for this study. For studies involving human research participant data or other sensitive data, we encourage authors to share de-identified or anonymized data. However, when data cannot be publicly shared for ethical reasons, we allow authors to make their data sets available upon request. For information on unacceptable data access restrictions, please see <http://journals.plos.org/plosone/s/data-availability#loc-unacceptable-data-access-restrictions>. | The authors have provided the data and use **DRYAD to create its DOI.**  DOI: 10.5061/dryad.h44j0zq0c |
|  | Please upload a new copy of Figure 2 as the detail is not clear. Please follow the link for more information: https://blogs.plos.org/plos/2019/06/looking-good-tips-for-creating-your-plos-figures-graphics/ | The authors have provided Figure 2 more clearly. |
|  | If the reviewer comments include a recommendation to cite specific previously published works, please review and evaluate these publications to determine whether they are relevant and should be cited. There is no requirement to cite these works unless the editor has indicated otherwise. | The authors have explained and revised the manuscript based on the reviewers’ suggestions. |

| **No.** | **Reviewer 1’s comments** | **Authors’ explanations** |
| --- | --- | --- |
|  | This article aims to explore the effect of User-Generated Content (UGC) on the purchasing behavior of environmentally friendly products in the Hospitality and Food Service Industry. It is an interesting and valuable field of research. However, how can the author say that this study uses a strong foundation, especially regarding the TPB? The author only applies one construct from the TPB, namely attitude. Furthermore, the implications of the study are not clearly presented, both theoretically and practically.  There is a lack of adequate foundation in the literature, and the definition of basic concepts is neglected. In addition, previous research supporting the hypothesis is also inadequate. | The authors would like to thank the reviewer for this valuable comment. We have revised the section of TPB:  The authors provide a clearer justification for applying the Theory of Planned Behavior (TPB) as a conceptual foundation. Although the study primarily focuses on attitude construct, this is theoretically justified because attitude represents the central determinant of behavioral intention within TPB (Ajzen, 1991). Prior studies on green consumer behavior have also applied partial models of TPB emphasizing attitude and intention when contextual factors are attitudinal or affective in nature (Han & Kim, 2010; Shin et al., 2018).  In the revision, we have explicitly clarified that TPB serves as the underlying theoretical lens explaining how *user-generated content (UGC)* shapes consumers’ pro-environmental attitudes, which in turn influence their purchase behavior. Additionally, to strengthen the theoretical framework, **Signaling Theory** (Spence, 1978) and **Brand-Trust Theory** (Morgan & Hunt, 1994) were integrated to explain information credibility and trust mechanisms within UGC contexts.  In this study, the author does not apply the entire TPB framework but only inherits a part, specifically the component “attitudes towards behavior”, which is the central element in the TPB model. This component explains how UGC influences consumers’ attitudes toward green products, thereby shaping their intention and behaviour to purchase environmentally friendly products.  Emphasizing the “attitude” factor has a scientific basis, because studies on green consumer behavior show that this is an important premise in forming behavioral intentions (Han & Kim, 2010; Shin et al., 2018). It is believed that positive attitudes towards the environment are expected to promote the perception that choosing green products is the right action, stemming from moral values and social benefits (Chen & Tung, 2014; Paul et al., 2016). This is consistent with TPB theory, which suggests that positive perceptions are closely related to behavioral intentions.  In addition to TPB, this study also incorporates Signaling Theory (Spence, 1978) and Brand-Trust Theory (Morgan & Hunt, 1994) to create a more reliable theoretical foundation. While TPB explains the mechanism of behavioral intention formation through attitude, Signaling Theory explains how online signals such as UGC help consumers evaluate the authenticity of information about green products (Erdem & Swait, 2001; Wilden & Gudergan, 2015). At the same time, Brand-Trust Theory introduces the concept of brand trust, demonstrating that trust plays a moderating role in the relationship between attitude and green behavioral intention (Chaudhuri & Holbrook, 2001; Delgado-Ballester & Munuera-Alemán, 2001). Based on this integration, the research model achieves higher generalizability and overcomes the limitation of only relying on a part of TPB. |
|  | The methods section is well-designed, but the rationale for choosing PLS-SEM over other software for this study needs to be clarified. | The authors have explained why PLS-SEM is used compared to others.  The PLS-SEM method was chosen because the research model is exploratory, includes both mediating and moderating variables, and focuses on predicting the relationship between latent variables. This method does not require data to follow a normal distribution, is suitable for small to medium sample sizes, and allows for the simultaneous assessment of direct, indirect, and moderating effects. The research model includes four main groups of factors: (1) UGC as the independent factor, (2) Environmental Attitude, concerns as the mediating factors, (3) Brand Reputation acts as a moderator, and (4) Purchasing Behavior of Environmentally Friendly Products as the dependent factor. The use of PLS-SEM enables practical testing of the influence mechanism of UGC on the purchasing behavior of environmentally friendly products in the hotel and catering industry. |
|  | The results of the study are presented clearly and logically, and the analysis is well-grounded in the methodology used. The article effectively demonstrates how UGC influences the environmental concerns, attitudes, and the intention to purchase green products. As well as the moderating role of brand reputation. Authors can add notes in each table for variable abbreviations. The conclusions are consistent with the earlier parts of the article and effectively combine theory with empirical data. The only possible improvement could be a deeper comparison of the results with previous studies in other cultural contexts. | The authors have added notes under the tables to clearly state what the variable abbreviations mean.  *Notes: BR is brand reputation; HVM is purchasing behavior for environmentally friendly products; LN is Environmental concerns; TD is Environmental Attitude; UGC is User-generated content; YD is Intention to purchase environmentally friendly products.*  The authors have made a comparison between our findings and empirical studies.  The results show that, first, UGC directly affects LN: a 0.601 positive coefficient shows that increased levels of user-generated content are associated with significant increases in environmental concerns. … These results match those from (Oliveira & Casais, 2019; Racherla & Friske, 2012; Yan et al., 2015). Restaurant chains are usually less environmentally sustainable (Madanaguli et al., 2022; Sakaguchi et al., 2018) due to the tremendous environmental impact (Kasim & Ismail, 2012) of which they generated large amounts of food and plastic waste and emissions as well as consumed excess water and energy.  Second, … In addition, hypothesis 2 is confirmed in the above analysis, which means that UGC positively influences environmental attitudes at Hospitality and Food Service Industry in Vietnam, especially in Ho Chi Minh City, as demonstrated by (Kang et al., 2014; Sultan et al., 2021; Yan et al., 2015) who demonstrate that consumers use UGC to assess the environmental aspects of a restaurant, such as the use of eco-friendly products or sustainable practices. By contributing feedback and sharing reviews about these experiences, UGC helps build the restaurant's reputation for sustainability. This reinforces consumers' positive attitudes toward the restaurant and influences their purchasing behavior toward environmentally friendly products and services. On the platform of the (Sultan et al., 2021) study, UGC fosters positive environmental attitudes by promoting emotional engagement with environmental issues. Greater exposure to environmentally focused UGC aligns individual attitudes with responsible environmental behavior.  Third, the path of UGC to HVM shown in Table 7 indicates a small but positive impact, with a coefficient of 0.180. …. The results are aligned to Bahtar & Muda (2016); Horst et al. (2007); Racherla & Friske (2012); Yan et al. (2015).  Considering the path between LN and HVM (purchase behavior), the relationship between LN and HVM demonstrates a moderate positive effect, with a path coefficient of 0.154. … Also, the evidence representing hypothesis 5 confirms that environmental concerns significantly influence purchasing environmentally friendly products at Hospitality and Food Service Industry in Vietnam, as mentioned by Balaskas et al. (2023); Kim & Choi (2005); Mostafa (2009); Yadav & Pathak (2016).  The next relationship between YD and HVM demonstrates a moderate positive effect, … This relationship is confirmed by Y.-S. Chen & Chang (2012); Hassan et al. (2016); Li & Nuangjamnong (2022); Thi Tuyet et al. (2024).  *Significantly, the findings confirm that brand reputation moderates the relationship between UGC and environmental concern; UGC and environmental attitude; intention to purchase environmentally friendly products and purchasing behavior.*  The findings indicate that brand reputation negatively moderates the relationship between UGC and environmental concern (LN) (Effect size: -0.133, T-statistic: 4.047, p = 0.000). This suggests that consumers rely less on UGC for environmental concerns when engaging with reputable brands, whereas lesser-known brands benefit more from UGC-driven consumer awareness. The results align with Signaling Theory, which posits that brand reputation reduces information asymmetry, making consumers less dependent on peer-generated content (Borah & Tellis, 2016; Erdem & Swait, 2001). Similarly, Brand-Trust Theory highlights that strong brands enhance consumer confidence in sustainability claims, diminishing UGC’s influence (Huang et al., 2021). …  The findings indicate that brand reputation negatively moderates the impact of user-generated content (UGC) on environmental attitudes (Effect size: -0.151, T-statistic: 2.936, p = 0.003). … Additionally, Brand-Trust Theory suggests that trust mitigates perceived risks, further weakening the influence of UGC when a brand’s reputation is strong (Grewal et al., 2020). Unlike previous studies (Afridi et al., 2021; Trivedi et al., 2018), which emphasized UGC’s universal influence on environmental attitudes, our results highlight that this effect is conditional on brand reputation….  The results show that brand reputation positively moderates the relationship between purchase intention (YD) and actual purchasing behavior (HVM) … These findings align with Expectancy-Value Theory, where brand reputation serves as a signal of trust and authenticity, reducing perceived risks and reinforcing consumer confidence. Empirical studies (Lau & Lee, 1999; Nguyen et al., 2018) confirm that reputable brands facilitate purchase decisions, while skepticism towards weaker brands hinders conversions (Leonidou & Skarmeas, 2017). For Vietnam’s hospitality industry, strong brands should leverage trust to promote eco-friendly products, while emerging brands must focus on transparency and engagement. This study contributes to Brand-Trust Theory (Morgan & Hunt, 1994) and sustainability marketing by highlighting how reputation strengthens the intention-behavior link. |
|  | The findings have direct implications only for businesses, emphasizing the importance of UGC to build trust regarding environmentally friendly products in the Hospitality and Food Service Industry. It is recommended to create new sections for each implication, both theoretical and practical, including limitations and suggestions for future research. | The authors have revised and added theoretical and practical, including limitations and suggestions for future research.  5.2. Theoretical implications and practical implications  This study extends the application of consumer behavior theories, including the TPB, Signaling Theory, and Brand-Trust Theory, in the context of the hotel and food service industry in Vietnam. The results show that UGC not only directly impacts environmental awareness and attitudes, but also indirectly promotes environmentally friendly purchase intentions and behavior. In particular, brand reputation is identified as an important moderator, helping to explain the difference in the level of consumer reliance on UGC between strong and weak brands. This finding contributes to strengthening the Signaling Theory by demonstrating that a high brand reputation helps reduce information asymmetry and mitigates the impact of UGC. At the same time, the study also complemented the Brand Trust Theory by demonstrating that brand trust and reputation can strengthen the link between green product purchase intention and behavior.  In practical, the research results provide valuable insights for developing sustainable marketing strategies in the hotel and catering industry. Major brands need to continue to maintain and strengthen their green image through official communication on environmental responsibility to increase customer trust. Meanwhile, less established brands should effectively leverage UGC, such as customer reviews, comments, and shares, to enhance their reputation and build trust. In addition, cooperating with environmental influencers or encouraging customers to post green reviews are also practical solutions to increase trust, form positive attitudes, and promote the intention to buy environmentally friendly products.   5. **Limitations and future research**   The study was conducted at 5-star restaurants in Ho Chi Minh City, so the results may not fully reflect other market segments or international contexts. Future studies can expand the scope of the survey to popular restaurant groups or food service chains in other areas to test the generalizability of the model. Furthermore, the various forms of UGC, such as video reviews, blogs, or influencer content, should be further considered to assess differences in their influence on consumer perceptions and behaviors. In addition, integrating additional variables such as “green trust”, “green perceived value”, or “subjective norm” into the model will help expand and strengthen the TPB theoretical framework, contributing to clarifying the impact mechanism of UGC on the purchasing behavior of environmentally friendly products. |

|  | **Reviewer 2’s comments** | **Authors’ explanations** |
| --- | --- | --- |
|  | Availability of the Data: Athough the authors mention the availibility of the data, it is available only by request, which is contrary to the policy of unrestricted data availibility of PLOS ONE. Suggestion: The authors must either upload the data to a public repository, or provide a reasonable justification for their inability to provide the data in full. | The authors have provided raw data and submitted online system, based on the requirements of journal. |
|  | Sample Representation: Though a sample of 331 participants is sizable, drawing all participants exclusively from five-star dining establishments in Ho Chi Minh City may be too limiting to reflect the entire consumer base in Vietnam. Suggestion: Including participants from a wider array of dining establishments and across different geographic areas in Vietnam would enhance the applicability and usefulness of the results. | In this study, the authors deliberately selected five-star restaurants in Ho Chi Minh City to ensure a uniform level of service quality and a group of customers with a high awareness of sustainable consumption. However, we agree that limiting the survey sample to a high-end segment and a single geographical area may affect the ability to generalize the results to all consumers in Vietnam. Therefore, in future studies, the authors will expand the scope of the survey to many different types of dining establishments (mid-range restaurants, cafes, green food chains) and other regions across the country to enhance the representativeness and practical application value of the research results.  Conducting surveys with customers at high-end restaurants helps ensure uniformity in service quality, experience, and awareness levels related to sustainable consumption. However, the authors found that the survey scope at 5-star restaurants in Ho Chi Minh City would result in a research sample that does not fully represent the characteristics of all Vietnamese consumers, thereby limiting the generalizability of the research results. Therefore, in subsequent studies, it is necessary to expand the scope of data collection to include different types of dining establishments (such as 3–4-star restaurants, cafes, or green dining chains) and more diverse geographical areas across the country to improve the generalizability and applicability of the results. |
|  | Practical Implication: While the focus in the manuscript on the theoretical part is pretty reasonable, it is the practical approach in relation to the business strategy which seems to be missing in this case. Suggestion: The authors must come up with strategies focused on the use of UGC to elevate brand image and positively influence consumer buying behaviour on green products, as well as practical strategies for the business. | The authors have revised and added theoretical and practical, as well as practical strategies for the business.    5.2. Theoretical implications and practical implications  This study extends the application of consumer behavior theories, including the TPB, Signaling Theory, and Brand-Trust Theory, in the context of the hotel and food service industry in Vietnam. The results show that UGC not only directly impacts environmental awareness and attitudes, but also indirectly promotes environmentally friendly purchase intentions and behavior. In particular, brand reputation is identified as an important moderator, helping to explain the difference in the level of consumer reliance on UGC between strong and weak brands. This finding contributes to strengthening the Signaling Theory by demonstrating that a high brand reputation helps reduce information asymmetry and mitigates the impact of UGC. At the same time, the study also complemented the Brand Trust Theory by demonstrating that brand trust and reputation can strengthen the link between green product purchase intention and behavior.  In practical, the research results provide valuable insights for developing sustainable marketing strategies in the hotel and catering industry. Major brands need to continue to maintain and strengthen their green image through official communication on environmental responsibility to increase customer trust. Meanwhile, less established brands should effectively leverage UGC, such as customer reviews, comments, and shares, to enhance their reputation and build trust. In addition, cooperating with environmental influencers or encouraging customers to post green reviews are also practical solutions to increase trust, form positive attitudes, and promote the intention to buy environmentally friendly products.  In addition, the research results also suggest some specific strategic directions to help businesses effectively exploit UGC to enhance green brand image and promote positive shopping behavior. Specifically, businesses can build online platforms that encourage customers to share green consumption experiences, while implementing proactive feedback mechanisms to increase engagement. In addition, integrating UGC content into brand communication strategies – such as citing real reviews in promotional campaigns or organizing “UGC campaigns” to spread sustainable values – will help strengthen credibility and increase consumer trust. Finally, businesses should combine UGC with customer behavior data to analyze green consumption trends, thereby adjusting marketing strategies more flexibly and effectively. |
|  | Theoretical Framework and Literature Review: Having a strong theoretical foundation is great, but the literature review needs more explanation on the application of such theories on the hospitality market in the like of Vietnam. Recommendation: The authors should add additional literature that has analyzed the impact of UGC on consumer behavior in the hospitality industry in emerging economies. | The authors have added the following paragraph into section of *“****2.1.1. User-generated content (UGC) and consumer behavior****”* to explain more the application of such theories on the hospitality market in the like of Vietnam.  In the context of the hotel and food service industry, especially in emerging economies, UGC plays an important role in shaping customer perceptions and decisions. Many studies have demonstrated that online reviews, images posted and shared by customers on social media have a significant impact on the intention to choose a restaurant or hotel (Litvin et al., 2018; Park et al., 2021; Reza Jalilvand & Samiei, 2012). In developing countries like Vietnam, according to (Kumar et al., 2018; Sang et al., 2024), UGC becomes a reliable source of information, helping users minimize risks in the purchase decision process because consumers do not fully trust information created by traditional media channels. Particularly, in the food/food industry, customer-posted reviews, experiences, and photos are a trusted source of information from the community, helping consumers evaluate the value of services and verify businesses’ “green” commitments by increasing transparency, authenticity, and trust (Behare & Babar, 2026). Therefore, to clarify the role of cultural factors, brand trust, and sustainable consumption motivation in consumer decision-making, it is necessary to study the impact of UGC on green products and services purchase behavior in emerging markets such as Vietnam. |
|  | Hypothesis Development: The authors did a great job in formulating the hypotheses but additional justification is a must for why the brand reputation is a strong moderator on the impact of UGC toward consumer behavior in the hospitality sector. Recommendation: The authors should explain in more detail why UGC is likely to have more impact on purchasing behavior on high reputation brands compared to low reputation brands. | The authors would like to thank the reviewer for this valuable comments. The authors have revised this section based on the reviewers’ suggestions.   2.1.1.1. *In the relationship between UGC and environmental concerns*   Signaling theory (Spence, 1978) explains how companies use trust signals, such as brand equity and CSR initiatives, to establish credibility. Well-known brands accumulate these signals over time, reducing consumer reliance on UGC to assess their environmental commitment. In contrast, lesser-known brands lack strong credibility signals, making consumers more dependent on UGC for evaluating sustainability claims. Therefore, brand reputation is expected to moderate the relationship between UGC and consumer environmental concern.  … This is especially proper in the hospitality industry, where it is challenging for customers to directly assess “green” factors, such as the sources of raw materials or operating procedures. For highly reputable brands, the brand name and image reduce the need for verification through UGC. Conversely, for less well-known brands, UGC becomes an objective confirmation channel for consumers to evaluate environmental commitment and service quality.   2.1.1.2. *In the relationship between UGC and environmental attitude*   ….  In the hospitality and food industry, brand reputation is not only the public image of the business but also the commitment to service quality, food safety and sustainable development orientation (Moon et al., 2022). A strong brand helps customers feel more secure and confident, reducing concerns about risks when choosing a service. (Erdem & Swait, 2004) argue that when consumers are familiar with a brand, they see it as a guarantee of quality, which reduces the need to search for further information. Therefore, in the case of strong brands, UGC often only plays a role in reinforcing trust rather than being a determinant of choice behavior, because brand reputation is enough to convince customers (X. Li et al., 2022; Vermeulen & Seegers, 2009). In contrast, for low-reputation or new brands, consumers lack the basis to evaluate the reliability of brand information, so they tend to rely more on other people's reviews and real-life experiences on social networks (online reviews, ratings, feedback). The results are supported by Signaling Theory and Brand Trust Theory, as well as empirical studies by (Filieri et al., 2018; Xie et al., 2014). Accordingly, UGC has a stronger impact on new or less well-known brands, because consumers rely on others' reviews to assess trustworthiness and reduce risk. In contrast, strong brands build trust through accumulated reputation, while weak brands rely on UGC to reinforce trust (Y.-S. Chen & Chang, 2013; Keller, 2016). |
